# GenGraph: a python module for the simple generation and manipulation of genome graphs

**DOI:** 10.1186/s12859-019-3115-8

**Published:** 2019-10-25

**Authors:** Jon Mitchell Ambler, Shandukani Mulaudzi, Nicola Mulder

**Affiliations:** Wellcome Centre for Infectious Diseases Research in Africa and Institute for Infectious Diseases and Molecular Medicine, University of Cape Town, Anzio Road, Cape Town, 7700 South Africa

**Keywords:** Genome, Graph, Toolkit, Python, Module

## Abstract

**Background:**

As sequencing technology improves, the concept of a single reference genome is becoming increasingly restricting. In the case of *Mycobacterium tuberculosis*, one must often choose between using a genome that is closely related to the isolate, or one that is annotated in detail. One promising solution to this problem is through the graph based representation of collections of genomes as a single genome graph. Though there are currently a handful of tools that can create genome graphs and have demonstrated the advantages of this new paradigm, there still exists a need for flexible tools that can be used by researchers to overcome challenges in genomics studies.

**Results:**

We present GenGraph, a Python toolkit and accompanying modules that use existing multiple sequence alignment tools to create genome graphs. Python is one of the most popular coding languages for the biological sciences, and by providing these tools, GenGraph makes it easier to experiment and develop new tools that utilise genome graphs. The conceptual model used is highly intuitive, and as much as possible the graph structure represents the biological relationship between the genomes. This design means that users will quickly be able to start creating genome graphs and using them in their own projects. We outline the methods used in the generation of the graphs, and give some examples of how the created graphs may be used. GenGraph utilises existing file formats and methods in the generation of these graphs, allowing graphs to be visualised and imported with widely used applications, including Cytoscape, R, and Java Script.

**Conclusions:**

GenGraph provides a set of tools for generating graph based representations of sets of sequences with a simple conceptual model, written in the widely used coding language Python, and publicly available on Github.

**Electronic supplementary material:**

The online version of this article (10.1186/s12859-019-3115-8) contains supplementary material, which is available to authorized users.

## Background

Modern genomics relies heavily on the use of a reference genome for common processes like variant calling, gene expression analysis, and even genome assembly. This reference sequence is often a consensus from a set of sequences that collectively represent anything from an individual isolate such as *Mycobacterium tuberculosis* H37Rv, to an entire species, in the case of the human genome assembly GRCh38, and the use of this single reference introduces a number of biases. The reference may be missing genes from some strains resulting in them being ignored in a differential expression analysis, or contain chromosomal rearrangements resulting in the effect of an upstream variant being misinterpreted. In terms of genome storage, the current standard is as linear sequence stored in a fasta file. Although they have served their purpose up until now, in the age of pangenomes and microbiome studies these representations have become limiting in terms of the file space they occupy, the functionality they provide, and their ability to represent population scale variation. These challenges have led to a move towards genome graphs. Where a pangenome is a collection of sequences that represent all variation between individuals in a defined clade, a genome graph is a graph representation of a pangenome where the sequences can be represented as a De Bruiijn graph, directed acyclic graph, bidirected graph, or a biedged graph. This representation of genomes offers a myriad of advantages over the use of a single reference genome.

Graphs are not a new concept in genomics, and are used for tasks including the assembly of genomes and the alignment of reads. Now, tools like vg (variant graph) [1], PanTools [2] and the Seven Bridges genome graph toolkit (https://www.sbgenomics.com/graph/) allow for the creation and utilisation of genome graphs in genomics work flows, and GfaPy allows for the creating, parsing, and editing of GFA graphs using Python [3]. These tools are developing rapidly, and include features that take advantage of the graph structure, allowing for read alignment and variant calling using the graph genome as a reference. While these tools are highly capable, there still exists a need for the development of more toolkits for genome graphs as advocated by Paten *et. al.,* in a recent review that discusses these tools and the improvements they have brought to variant calling [4].

GenGraph is a genome graph creation and manipulation toolkit created in Python that focuses on providing tools for working with bacterial genome graphs within an initiative conceptual model. It is able to create a genome graph using multiple whole genomes and existing multiple sequence alignment (MSA) tools, allowing any current or future algorithm to be employed. In this article we outline the structure of the genome graph created by GenGraph, and the methods for its creation, and give examples of how the provided functions may be used to extract biologically interesting features from the graph. Further examples of applications are available on the project Github page.

## Implementation

GenGraph is written in Python, a widely used language in the biological sciences that is easy to learn, powerful, and has numerous useful libraries including Biopython, Numpy and NetworkX. GenGraph was implemented as both a Python tool and a module with modified NetworkX graph objects whose attributes may be accessed in the manner described in the NetworkX documentation.

### Structure of the graph

**Fig. 1:**
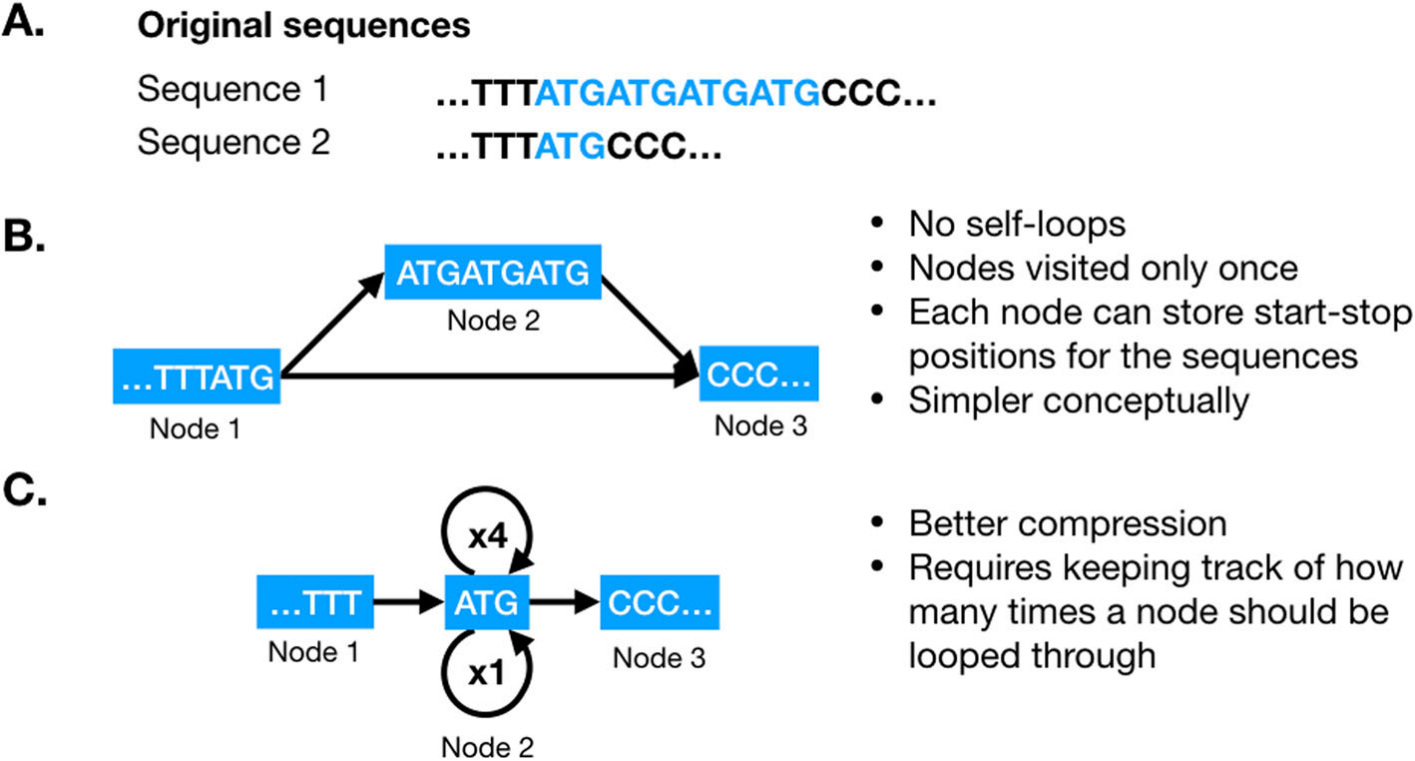
Representation of repeats in the genome graph. **a**, Two sequences where sequence 2 contains 3 additional “ATG” repeats high-lighted in blue. **b**, GenGraph represents only differences, with node 1 representing both sequences, node 2 representing the additional repeats found only in sequence 1, and node 3 the sequence that is once again shared. **c**, This is opposed to an approach where the “ATG” repeat is represented as a single node with a self loop. This approach may be neater and result in better compression, but raises many practical problems including not allowing the node to be labeled with the sequence start and stop positions

A GenGraph graph is a directed sequence graph, where the individual genomes are encoded as walks within the graph along a labeled path. Each node represents a sub-sequence that is homologous between the component sequences. This implies the sequences have a shared evolutionary origin, and are not just identical sequences, and biological representation of the genomes is prioritised over a compressed data structure (Fig. 1). This is an important design choice in that it allows for a more intuitive use of the graph, and a simpler conceptual model. As the graph contains no self loops, creating functions that require traversal is kept simpler.

**Fig. 2:**
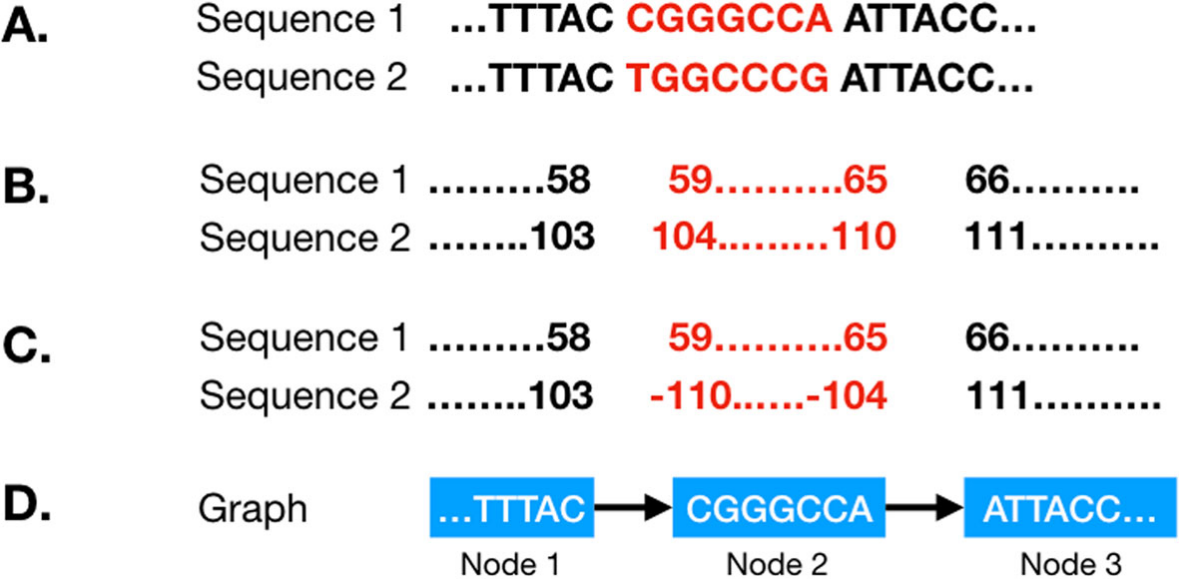
Representation of inversions in the genome graph. During the first step of genome graph creation, co-linear blocks are identified. In some cases, these may be homologous sequences that have been inverted. GenGraph represents these sequences in a single node (that may be broken down into more nodes in the second step) and represents the inverted state of the sequence by negative nucleotide position values in the node. **a**, Two sequences are shown where an inversion has taken place. This is normally a larger stretch of sequence perhaps a few kb in length. **b**, The positions of the sequences are different, as is generally the case with homologous sequences. The positions of the nucleotides flanking the breakpoints are shown. **c**, The inversion in the second sequence is represented by reversed negative nucleotide position values. **d**, This way, both sequences are represented in the same node, and to recreate sequence 2, the sequence in the node is simply reverse-complimented

The coordinate system relies on storing the relative start and stop positions for each component sequences in each node. This means that given only a single node, one can determine that the first ’A’ nucleotide in the node has position 132 in isolate A, 21,310 in isolate B, and so on. This allows for existing annotations to be used, and for the sequence of a gene for a particular isolate to be retrieved from the graph given a traditional GTF file, a common task for which a function has been created. The coordinate system also allows for inversions to be represented in a single node (Fig. 2). This results in a more intuitive representation of the relationship between the sequences as genes that fall within the inverted sequence are still found in the same node in both isolates, and the concept of a chromosomal breakpoint is represented by the edges either side of the node. Descriptions of the node and edge attributes can be found in Tables 1 and 2.

**Table 1:** Information on the node attributes used in a gengraph genome graph

Node attribute	Type	Description
name	String	A unique name identifying the node. When a node is split, the resulting nodes inherit the original node’s value appended with a new number. So if node Aln_66 is split into 4 nodes, they are named Aln_66_1, Aln_66 _2, Aln_66_3, Aln_66_4.
sequence	String	The nucleotide sequence that is represented by this node.
ids	String	This is a comma separated list of the isolates that are represented by this node.
(isolate)_leftend, (isolate)_rightend	Integer	For each isolate, the positions of the first and last nucleotides represented by the node is recorded. So for isolate H37Rv, H37Rv_leftend = 13,203.

**Table 2:** Information on the edge attributes used in a gengraph genome graph

Edge attribute	Type	Description
name	String	Edges are named by a combination of the two nodes that they link. Eg: Aln_48_50 (-) Aln_48_49 would be the name of the node that links nodes Aln_48_50 and Aln_48_49
ids	String	This is a comma separated list of the isolates that are represented by this edge.

### Creation of the genome graph

**Fig. 3:**
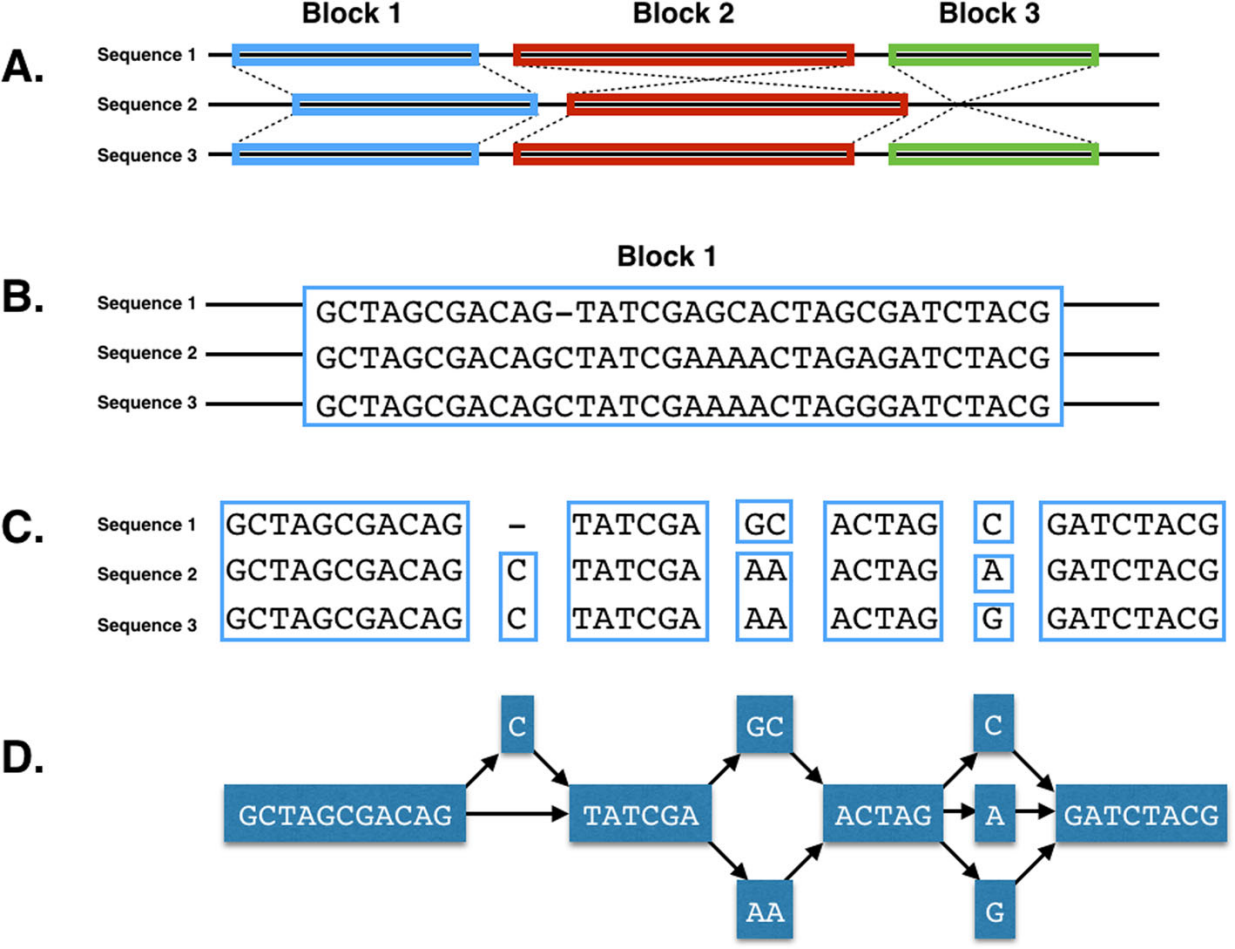
Overview of the GenGraph algorithm. **a**, Co-linear blocks of sequence are identified to determine the structural relationship of the sequences. **b-c**, Each block is then realigned using a MSA tool. **c-d**, Identical sequences are reduced into nodes and edges created

The graph is created as a modified Python NetworkX graph object, the details of which may be found here (https://networkx.github.io). GenGraph currently creates a genome graph in two steps (Fig. 3). First, large structural differences between the genomes including large deletions and chromosomal inversions are identified by finding large blocks of co-linear sequence between the genomes using a tool like progressiveMauve [5]. These represent regions of structural conservation, and are temporarily stored in a single node within the initial graph, even if they are imperfect alignments. GenGraph then realigns the sequences in these initial nodes using the selected MSA tool, and finds the best local alignment for the sequences. The relative start and stop positions for the region of aligned sequence contained within the node is stored for each of the isolates that are included in that node as attributes of the NetworkX node object. This is then converted into a sub-graph by collapsing shared regions into single nodes, and creating edges so that a path exists for each of the original sequences through the sub-graph. This sub-graph then replaces the initial temporary node from the initial structural graph.

The process of identifying the co-linear blocks and subsequent realignment is done using functions that wrap existing alignment tools. Currently Muscle [6], Mafft [7] and Clustal Omega [8] are supported for the local MSA. The final NetworkX graph objects created by GenGraph may be exported as GraphML, XML, or as a serialised object, though various other formats may be added in future. GenGraph creates a report file containing information such as the number of nodes and edges in the graph, the average in and out degree of the nodes, the total sequence length of all the nodes in the graph and the density of the graph (Additional file 1). This information can be used to monitor how graphs change as more genomes are added as well as the relationship between the number of features and the graph size.

### Available graph functions

GenGraph is available as both a command line tool, and a Python module. Both allow for the creation of a genome graph, and the use of an existing genome graph for downstream analysis.

These functions include simple processes like extracting a single genome in fasta format for a specific isolate, or extracting a sub-sequence, as well as more complicated functions that take advantage of the coordinate system to translate the position of a gene in one genome to its position in another.

To demonstrate the use of GenGraph, we downloaded the complete genome assemblies of various *Mycobacterium tuberculosis* isolates from the NCBI database (https://www.ncbi.nlm.nih.gov/genome/) and used them to construct a genome graph using progressiveMauve for the structure graph, and Clustal Omega for the realignment of the blocks. We then use the available functions and simple Python code to identify conserved regions of the genomes, compare the sequence of a gene between isolates, and visualise a variant in a gene.

## Results and Discussion

### Structure of the graph

GenGraph creates the genome graph based on whole genome sequence alignments that are conducted in two parts. First, the identification of large co-linear blocks, then the realignment of those blocks. This allowed for the creation of a genome graph containing multiple bacterial genomes, including MTB isolates like W-148 that contain large chromosomal rearrangements [9]. The genome graphs are thus able to capture all variants from large scale structural differences between isolates such as chromosomal rearrangements, down to smaller scale differences including SNPs and copy number variations. The structure aims to represent a biologically accurate representation of the evolutionary relationships between the sequences of the different isolates. In doing so, it maintains a simple conceptual model, which makes interpreting the graph simple, and in turn helps developers to create new tools easier.

### Global and local alignment: Performance

A primary feature of GenGraph is the use of existing MSA tools for the creation of the graph by wrapping the tools in functions. Because GenGraph uses these alignment tools to create the graph structure, users may use parameters or aligners that are best suited for the organism. With MSA being the current speed bottleneck in the creation of genome graphs, the scalability of GenGraph is dependent on the ability of these alignment tools. This allows the toolkit to evolve and improve with time, as well as utilise alignment tools that are best suited to the dataset at hand, and adapt to new innovations including GPU acceleration or field-programmable gate array chips.

Graph generation runtime increases in a linear fashion, influenced by the number of sequences being aligned, their length, and similarity. By breaking down the genomes into partially pre-aligned blocks, GenGraph is able to align multiple long genomes in segments and with the current version of mafft able to align up to 30,000 sequences in a block.

GenGraph was able to create a genome graph containing 5 MTB genomes on a 2012 i7 Macbook Pro with 8 GB ram using Mafft in 53 min and 10 genomes in 2 h and 44 min (Additional file 2). For smaller genomes, 300 HIV-1 genomes were aligned and converted to a genome graph in 35 min. Currently GenGraph does not take advantage of multiprocessing, an enhancement that will be made in an upcoming update. From testing we see the scalability of GenGraph is dependent on the power of the latest alignment tools, the number of sequences being aligned, their length, and their similarity, though in general we observe a linear increase in genome graph generation time as the number of sequences increase (Table 3). Graph generation represents the most computationally intense and time consuming process, while downstream analysis benefits from the use of the data in an aligned form.

**Table 3:** An increase in file size was observed per genome added to the graph that demonstrated the compression of data that occurs by collapsing regions of shared aligned sequences into single representative nodes

Number of genomes	1	2	3	4	5	6	10
File size	4,5Mb	5,9Mb	7,6Mb	8,5Mb	11Mb	13Mb	38Mb
Number of nodes	0	3,690	8,106	9,320	13,264	15,355	43,290
Number of edges	0	4,886	10,868	12,485	17,823	22,296	73,652

The compression is related to the similarity of the sequences, as sequences that only differ by few bases will only require a few additional nodes. (Additional file 3)

### Available graph functions & toolkit

The structure used by GenGraph and the provided functions makes writing code simple for anyone that is familiar to Python. While the toolkit provides options to create genome graphs and extract sequences using command line, the GenGraph module provides access to functions and methods that can be used to create new tools or conduct analyses.

#### Use case 1: Identifying conserved regions

The following code can be used to identify the largest uninterrupted sequence common to all the isolates in the provided genome graph. These conserved regions may contain core genes required for survival and are often drug target candidates.


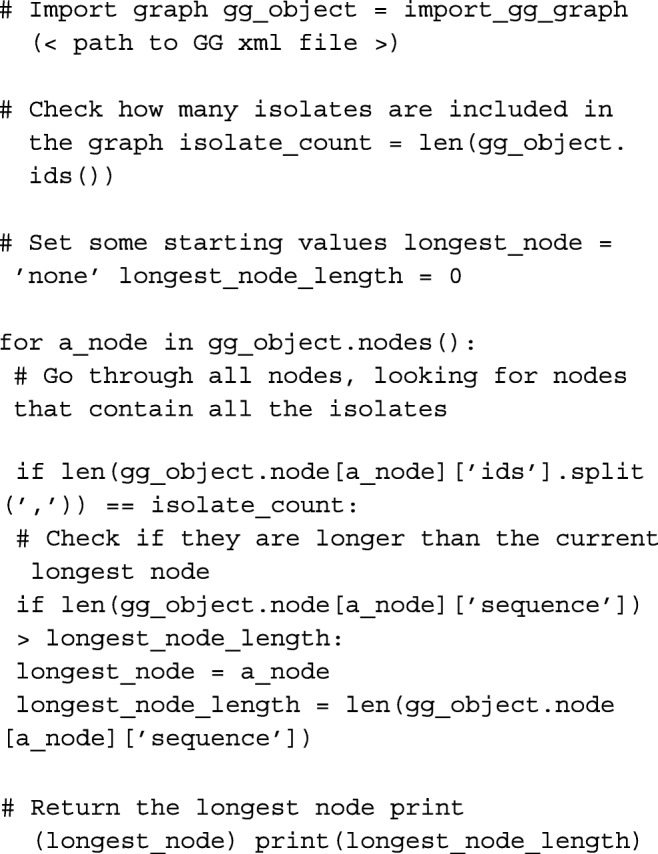


The inverse can be done, finding all nodes with length less than 3 bp and belonging to only one isolate. This will represent isolate specific SNPs that could be useful to explain unique characteristics, for example in the case of a genome graph composed of 10 harmless and one pathogenic strain. This process would be far more difficult using multiple vcf files and reference genomes, particularly if mutations are found in genes that are not found in the reference genome.

#### Use case 2: Comparing genes between isolates

A common task is the comparison of genes from different isolates, and describing how they differ. First we extract the carB gene in the MTB isolate H37Rv.


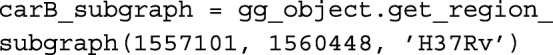


This subgraph contains the sequence of the carB gene. If the graph has only one node, and all of the isolates are found in the node ’ids’ list, then all of the isolates have the same gene with no mutations. A user could then iterate though all genes in an annotation file, and identify those conserved across all strains to identify a core genome. If there is more than one node, or not all isolates are represented in that node, further investigation can be done by adding another attribute to each node representing the sequence length, and then exporting the subgraph for visualisation in Cytoscape [10].


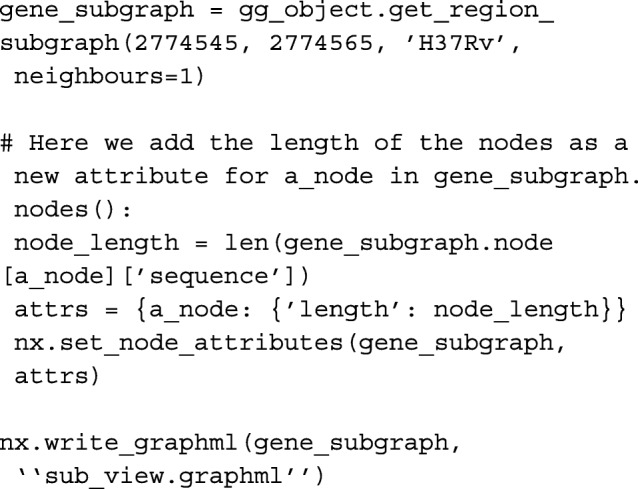


**Fig. 4:**
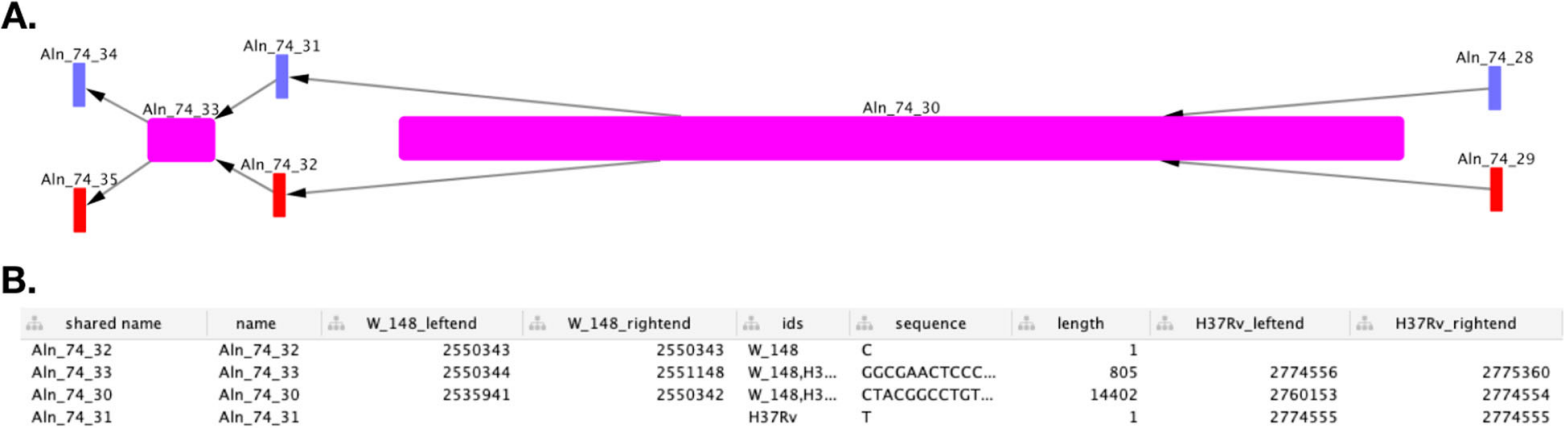
Plot of exported subgraph. **a**, Cytoscape allows for the styling of imported networks, and by mapping the node width to the sequence length it is simple to visualise which nodes represent insertions. Nodes can be coloured by which isolates they contain, in this case Beijing isolates were represented by red nodes, H37Rv by blue nodes, and purple nodes represent nodes shared by all isolates. **b**, For more detail on nodes of interest, a table listing the node and edge attributes is also available

With a few simple lines of Python, we are able to compare genes across different isolates, visualising interesting differences (Fig. 4). With more complex code, functions that calculate sequence similarity between isolates can be created, and allow for the identification of homologues to be done. This has been used in the creation of a homology matrix, which is useful for interpreting results from isolates with poor or missing annotations. The homology matrix created by GenGraph allows mapping of gene IDs between isolates to identify orthologues in a gene-order aware manner. As orthologues are identified by their relative position within in the graph, and not by their sequence similarities, the correct orthologues of genes with multiple paralogues such as the PE/PPE genes in *M. tuberculosis* are identified. Additional tools for cladogram construction and pan-transcriptome extraction have been created that take advantage of the genome graph structure in a similar manner and are outlined on the GenGraph GitHub page under the Wiki.

### Comparisons

GenGraph was made to be modular and built around the described genome graph structure. Although GenGraph currently uses a two stage graph genome creation pipeline the focus is not on assembly, support for cactus and de Bruijn graphs to be collapsed into the GenGraph structure will be provided in future releases, as well as importing of graphs created by vg. While cactus and de Bruijn graphs are useful for assembly and alignment, their structures are not intuitive to the majority of downstream users. The functionality provided by GenGraph provides support for the downstream use of genome graphs, and designed to make it easy for even novice programmers to start using genome graphs in their workflows, encouraging adoption and making the transition from fasta based reference thinking simpler. The structure of graphs used in GenGraph is most similar to the structure used in vg (https://github.com/vgteam/vg/wiki/Visualization) but differs in that vg is written in C++, and we believe a genome graph tool for Python would be more accessible for the research community.

## Conclusions

GenGraph brings genome graphs into the world of Python, with a toolkit that allows users to create genome graphs using MSA tools. It is able to scale from small viral genomes to bacterial genomes on desktop computers, with further testing for large genomes already underway. GenGraph uses external alignment tools for the creation of the alignments used in generating the genome graph, making it able to utilise any existing or future alignment tools to boost its performance. Because only existing graph file formats are used, the graphs can be imported and visualised by common tools, including Cytoscape and R.

To facilitate adoption, GenGraph includes a number of useful tools and functions in order to facilitate adoption into work flows, allowing users to quickly create code that carries out common tasks and analysis using genome graphs. Combined with an intuitive conceptual model and built for Python, one of the most widely used programming languages in the biological sciences, GenGraph provides a starting point for the development of a new generation of genome graph based tools.

## Availability and requirements

**Project name**: GenGraph**Project home page**: https://github.com/jambler24/GenGraph**Operating system(s)**: Platform independent**Programming language**: Python 3**Other requirements**: NetworkX, Mauve, and Muscle. A docker image is also available in the github repository containing all these requirements.**License**: GNU LGPL**Any restrictions to use by non-academics**: No

## Additional material

Additional file 1 Example report file for the genome graph created by GenGraph. The report file is in.txt format and contains details of the generated genome graph.

Additional file 2 Genome graph containing five MTB genomes. The graph was created from the assemblies of six MTB isolates (Beijing, C, CDC1551, F11, H37Ra, H37Rv) from GenBank, and saved in GraphML format. This file is viewable in Cytoscape and may be imported using Python’s NetworkX package.

Additional file 3 Graph showing the effect of sequence similarity on file size. To test the effect of different sequence similarities on the output file size, single base substitution mutations were simulated at different rates across 1 kb sequences. At 1 SNP per 1kb, there is only a slight increase in size as more sequences are added. This represents an upper estimate, as sequences were mutated independently, where as in related sequences some mutations would be shared and not require new nodes to be created. In the case of whole genomes, three closely related *Mycobacterium tuberculosis* KZN strains of 4.5 MB can be converted to a single 4.6 MB GraphML file.
