# Supplementary figures and images for: GenGraph: a python module for the simple generation and manipulation of genome graphs
Source: BMC Bioinformatics. 2019 Oct 25;20:519. doi: 10.1186/s12859-019-3115-8 (PMC6894214; doi:10.1186/s12859-019-3115-8)

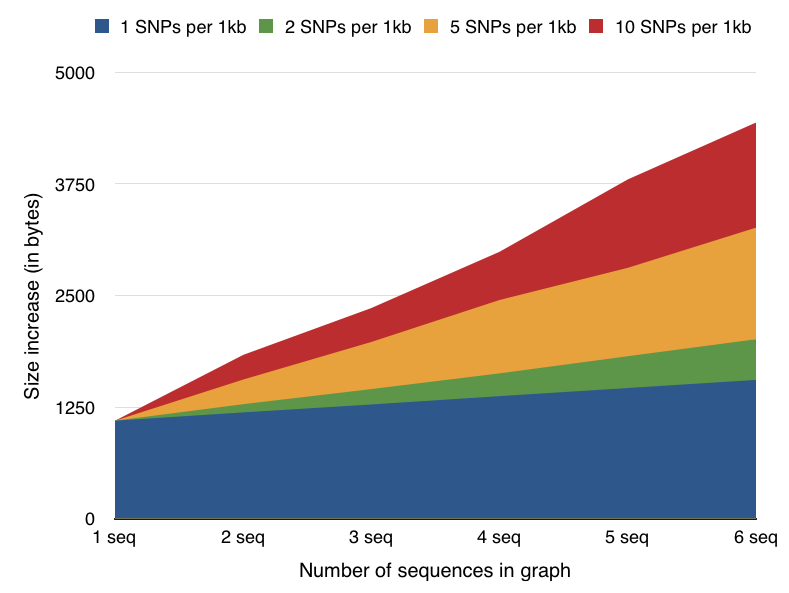

Supplement: Supplementary file 3 — Graph showing the effect of sequence similarity on file size. To test the effect of different sequence similarities on the output file size, single base substitution mutations were simulated at different rates across 1 kb sequences. At 1 SNP per 1kb, there is only a slight increase in size as more sequences are added. This represents an upper estimate, as sequences were mutated independently, where as in related sequences some mutations would be shared and not require new nodes to be created. In the case of whole genomes, three closely related Mycobacterium tuberculosis KZN strains of 4.5 MB can be converted to a single 4.6 MB GraphML file. [file 12859_2019_3115_MOESM3_ESM.png]
